# Acute Urticaria and Anaphylaxis: Differences and Similarities in Clinical Management

**DOI:** 10.3389/falgy.2022.840999

**Published:** 2022-04-15

**Authors:** Luis Felipe Ensina, Taek Ki Min, Mara Morelo Rocha Félix, Carolina Tavares de Alcântara, Célia Costa

**Affiliations:** ^1^Division of Allergy, Clinical Immunology and Rheumatology, Department of Pediatrics, Federal University of São Paulo, São Paulo, Brazil; ^2^Department of Pediatrics, Soonchunhyang University Seoul Hospital, Soonchunhyang University College of Medicine, Seoul, South Korea; ^3^Division of Allergy and Immunology, Department of General Medicine, School of Medicine and Surgery, Universidade Federal do Estado do Rio de Janeiro, Rio de Janeiro, Brazil; ^4^DermAlergo Clinic, Belém, Brazil; ^5^Immunoallergology Department, Hospital de Santa Maria, Centro Hospitalar Universitario de Lisboa Norte (CHLN), EPE, Lisbon, Portugal

**Keywords:** urticaria, angioedema, anaphylaxis, diagnosis, treatment

## Abstract

Acute urticaria is a common condition that presents with wheals and/or angioedema. However, these symptoms are also frequent in anaphylaxis, a life-threatening reaction that should be immediately diagnosed and treated. In both, mast cells play a central role in the physiopathology. Causes and triggers of acute urticaria and anaphylaxis are similar in general, but some peculiarities can be observed. The diagnostic approach may differ, accordingly to the condition, suspicious causes, age groups and regions. Adrenaline is the first-line treatment for anaphylaxis, but not for acute urticaria, where H1-antihistamines are the first choice. In this paper, we review the main aspects, similarities and differences regarding definitions, mechanisms, causes, diagnosis and treatment of acute urticaria and anaphylaxis.

## Introduction

Urticaria is a condition with a lifetime prevalence rate of up to 20% and characterized by the development of wheals, angioedema, or both (Table 1) (1, 2). Acute urticaria, which is defined by the occurrence of symptoms for up to 6 weeks, can be the only manifestation of a hypersensitivity reaction but can also be associated with other systemic symptoms, indicating an anaphylactic reaction (1, 3). This paper aims to review the mechanisms, triggers, diagnosis, and treatment of acute urticaria and anaphylaxis, highlighting the differences in managing both conditions.

**Table 1:** Clinical characteristics of urticaria [adapted from (1)].

**Typical features of a wheal:** 1. A sharply circumscribed superficial central swelling of variable size and shape, amost invariably surrounded by reflex erythema 2. An itching or sometimes burning sensation 3. A fleeting nature, with the skin returning to its normal appearance, usually within 30 min to 24 h
**Angioedema is characterized by** 1. A sudden, pronounced erythematous or skin-colored deep swelling in the lower dermis and subcutis or mucous membranes 2. Tingling, burning, tightness, and sometimes pain rather than itch 3. A resolution slower than that of wheals (can take up to 72 h)

## The Mast Cell and its Role in Urticaria and Anaphylaxis

Urticaria and anaphylaxis are often but not always related to mast cell activation from multiple triggers, including IgE-mediated and non–IgE-mediated mechanisms. Mast cell plays a broad critical role in the innate and acquired immune response because they express multiple receptors responding to specific antigens, as well as circulating complement components and fragments, immune complexes binding IgG and IgM, cytokines, changes in blood pressure, and immunologic activation (4, 5). Therefore, mast cell activation in patients with urticaria and anaphylaxis is more likely to occur through multiple pathways in addition to IgE.

Mature mast cells are primarily found in tissues where external pathogens enter the body, including the skin, gastrointestinal tract, and airway. Immunological staining of tissues has revealed two types of human mast cells characterized by their neutral protease content: mast cells which are tryptase-positive but chymase-negative (MCT), and mast cells which are both tryptase- and chymase-positive (MCTC) (6). MCT are found typically at mucosal tissues, such as the intestine, lung and nose, are T-lymphocyte dependent and are increased in number in allergic disease (7). In contrast, the development of MCTC is independent of lymphocytes, and they are located primarily in the skin and gastrointestinal submucosa. MCTC account for more than 99% of the mast cells in the dermis of both lesional skin and non-lesional skin of patients with chronic spontaneous urticaria (CSU) (8). Immunoglobulin E (IgE)-dependent stimulation leads to degranulation of both subtypes, but MCTC can also be activated by IgE-independent mechanisms.

Activation of mast cells occurs when allergen-specific IgE is bound by allergen and interacts with high-affinity IgE receptor (FcεRI) on their surfaces (9). In addition to FcεRI, human mast cells express receptors for IgG (FcγRII/III), complement (C3a/C5a), drugs [Mas-related G protein-coupled receptor X2 (MRGPRX2)], opioids, neuropeptides, nerve growth factor, stem cell factor and cytokines, ligation of which modify mast cell function-survival, maturation, differentiation, growth, apoptosis, and degranulation (5, 10). Mast cells can be activated through newly identified MRGPRX2 by fluoroquinolones such as ciprofloxacin, icatibant and general anesthetics such as atracurium, rocuronium, tubocurarine, independent of the IgE-FcεRI pathway (11, 12). In this pathway, binding of these drugs and drugs expressing the THIQ (tetrahydroisoquinoline) motif directly to MRGPRX2 results in protein kinase A and phosphoinositide 3-kinase pathway activation, calcium release and degranulation (5, 11, 12). Also, activation of mast cells through MRGPRX2 may contribute to neurogenic inflammation, pain, itch, and pruritic skin diseases, including CSU (13). Increased MRGPRX2 protein expression has been reported in the skin of patients with CSU (14).

## Causes and Triggers of Acute Urticaria and Anaphylaxis

### Acute Urticaria

Common causes or triggers of acute urticaria include infections (viruses, bacteria, and parasites), food and medicines and less frequently latex, Hymenoptera venom, vaccines, physical stimuli, which a detailed history should identify. The prevalence of different etiologies varies among different age groups. In half of the cases, it is not possible to identify a specific cause for acute urticaria, being classified as idiopathic (15–17).

Respiratory infections, mainly of viral etiology, are considered the most related trigger to acute urticaria in all age groups (about 40% in adults and 60% in children) in different populations (16, 18–23). Gastrointestinal and urinary tract infections are also associated. In a study with children, infection was the most frequently documented cause for acute urticaria (48.6%), followed by drugs (5.4%), and food allergies (2.7%) (24). In the pediatric age, herpes virus (especially cytomegalovirus, Epstein-Barr virus, and herpes virus type 6) was the principal agent responsible for acute and recurrent flares of urticaria. Other viruses, including adenovirus, rotavirus, parvovirus B19, respiratory syncytial virus, and recently SARS-Cov2, have also been described as potential triggers (25). *Mycoplasma pneumoniae* and *Streptococcus spp* are frequent, while Chlamydia is less reported as an acute cause. Parasites may also induce acute urticaria with eosinophilia (15). In adults, hepatitis viruses (A, B and C) are most frequently implicated in acute urticaria (25).

However, the prevalence of infectious causes tends to reduce with age, and drug therapy with antibiotics (beta-lactams) and non-steroidal anti-inflammatory drugs (NSAIDs) often trigger urticaria in infants and children. At the same time, NSAIDs, angiotensin-converting enzyme inhibitors and neuromuscular blockers are more implicated as potential triggers of acute urticaria in adults (26).

Food allergies are minor causes of acute urticaria (16, 24, 27). The most implicated food allergens are cow milk, eggs, peanuts, tree nuts, wheat, and seafood (16, 18–23). Certain foods such as some types of fish (tuna, sardines, anchovies), cheeses (Emmental and gouda), salami, sausage, fruits (strawberry), vegetables (especially tomatoes) and beverages (wine and beer) have been described as triggers of recurrent urticarias, especially in patients intolerant to histamine or with deficiency of the enzyme diamine oxidase, responsible for histamine degradation. However, predicting the benefit of low histamine diets is practically impossible due to different dietary habits worldwide, and more studies on the subject are needed (28, 29).

Urticaria caused by latex, Hymenoptera venom and vaccines are less frequent. However, hypersensitivity to insect bites in Latin America countries is described as the main inducer of urticaria in children (30). Physical stimuli (dermographism, increased body temperature and cold) rarely cause acute urticaria, especially in children (15).

### Anaphylaxis

Similar to urticaria, the profile of anaphylaxis triggers depends on age and different geographic areas. Moreover, in up to 35% of anaphylaxis cases, a specific trigger may not be identified during the acute event or in subsequent evaluations, characterizing an idiopathic picture (31–39).

Worldwide, food, insect venom and drugs are the most frequent triggers (40–44). Food is the most common trigger for severe anaphylactic reactions in children, while drugs and insect venom are common triggers in adults (40, 42, 44–46).

In young children, due to the greater need for hospitalization, anaphylaxis from food and drugs is notably greater. In infants and young children, food, especially cow milk, eggs, peanuts, tree nuts, sesame and wheat, are the most common causes of anaphylaxis (41–44, 46, 47). Nuts, cashews, and hazelnuts are also causes of anaphylaxis in school children. Food dyes are not a common cause of food allergy (18).

Food-induced anaphylaxis in adults varies by region and food exposure. In North America and Australia, peanuts and nuts are the main triggers for anaphylaxis, while shellfish are most often associated in Asia. In central Europe, the foods most associated with anaphylaxis are peanuts, tree nuts, sesame, wheat, and shellfish. However, in southern Europe, it is lipid transfer proteins (pan-allergens responsible for cross-reactivity between fruits, vegetables, and pollens) associated with cofactors that are the most frequent food allergens. Sesame seed and buckwheat are common causes of anaphylaxis in the Middle East and Korea, respectively (3, 41–47).

Less common allergens that can trigger late anaphylaxis reactions such as alpha-gal should also be investigated (3).

Medications are also a cause of anaphylaxis, and reactions usually appear in school-age children and adolescents. They are found to be the most common cause of anaphylaxis-related deaths both in adults and in children in different countries, but this may vary depending on the method of the study and database searched (18, 31, 48–50).

Antibiotics, particularly beta-lactams, are described as the main triggers of drug-induced anaphylaxis in childhood, with few reports of anaphylaxis to other non-beta-lactam antibiotics, such as macrolides. In adults, penicillin, cephalosporins, and sulfonamides are the most implicated antibiotics (48, 51–58).

Non-steroidal anti-inflammatory drugs (NSAIDs) are the second leading cause of drug-induced anaphylaxis in children worldwide. However, in Latin America, NSAIDs are the first cause in both children and adults (56, 59). In addition to antibiotics and NSAIDs, neuromuscular blockers, anesthetics, opioids, hypnotics, ethylene oxide, plasma expanders, and dyes (patent blue and methylene blue) have been frequently involved in perioperative anaphylaxis (3, 22). In some countries latex allergens remain a significant trigger of perioperative anaphylaxis (60, 61). But the incidence of latex allergy has decreased in many places due to primary prevention measures such as wearing powder-free latex gloves and latex-free surgical material in the operating room (62–64). Reactions to radiographic contrast media have occurred less frequently with the use of non-ionic and low osmolality contrasts rather than with monomeric ionic (65).

New triggers have been identified as a cause of anaphylaxis and include immunobiological drugs, chemotherapeutics, chlorhexidine, polyethylene glycol, and methylcellulose. In general, medications are the leading cause of fatal anaphylaxis in adults and children (3).

In the United States, antibiotics, NSAIDs, immunomodulators and biologic agents are the most implicated agents in drug-induced anaphylaxis, whereas, in the United Kingdom, general anesthetics are frequently associated with fatal drug-induced anaphylaxis (32).

Insect venom-induced anaphylaxis also exhibits regional patterns. Bee venom is the most frequent trigger in South Korea, and it is also more frequent in children. While in central Europe, the wasp is the insect that induces the most anaphylaxis. In other regions, such as America, Asia and parts of Australia, and venom is an important trigger of anaphylaxis. Fatal cases of anaphylaxis from insect venom are most associated with adults (3).

Exercise-induced anaphylaxis and anaphylaxis induced by food-dependent exercise are two rare but significant entities. Various activities such as yard work, walking and running can trigger an exercise-induced anaphylaxis condition. Symptoms can occur during or after physical activity, but it is usually challenging to predict crises. In induced anaphylaxis by food-dependent exercise, symptoms occur when the causative food, such as seafood, dairy products, and wheat, is consumed minutes to several hours before exercise. In these cases, patients should avoid eating these foods 4–6 h before exercise (18).

Some external cofactors or associated conditions play an important role in the development of allergic reactions, including anaphylaxis. In the presence of cofactors such as physical exercise, drugs (e.g., nonsteroidal anti-inflammatory drugs, proton pump inhibitors), acute infections, alcohol and menstruation, allergic reactions may be elicited at lower doses or there may be more severe or life-threatening clinical reactions (40). There are associated conditions that work as cofactors jeopardizing patients, or increasing mortality (e.g., unstable asthma, mast cell disorders, cardiovascular diseases). However, the mechanism of action of such cofactors have not been fully identified yet, but increased bioavailability of allergen due to increased intestinal permeability and intestinal allergen absortion, decreased activation threshold on the cellular level and transient plasma hyperosmolality, are among the potential mechanisms proposed (40, 66, 67).

Supposedly, cofactors play a role in approximately 14–30% of anaphylactic reactions. Therefore, in a given patient these cofactors should always be considered in the clinical history and eliminated when possible, to reduce the risk of a future severe reaction (66, 68).

## Diagnostic Approach for Acute Urticaria and Anaphylaxis

### Urticaria as a Manifestation of Anaphylaxis

Anaphylaxis is a serious allergic reaction that is rapid in onset and can be fatal. Skin and mucosal manifestations are frequent but not always present (69). Anaphylaxis is highly likely when any one of three criteria are fulfilled (Table 2) (3, 32, 70, 71).

**Table 2:** Clinical criteria for diagnosing anaphylaxis [adapted from (67)].

**Anaphylaxis is highly likely when any of the following three criteria is fulfilled:**
**Acute onset of an illness (minutes to several hours) with involvement of the skin, mucosal tissue or both and at least one of the following** a. Respiratory compromise b. Reduced BP or associated symptoms of end-organ dysfunction
**Two or more of the following that occur rapidly after ex-posure to a likely allergen for that patient (minutes to several hours):** a. Involvement of the skin–mucosal tissue b. Respiratory compromise c. Reduced BP or associated symptoms d. Persistent gastrointestinal symptoms
**Reduced BP after exposure to known allergen for that patient (minutes to several hours):** a. Infants and children: low systolic BP (age specific) or >30% decrease in systolic BP {Low sys-tolic blood pressure for children is defined as <70 mmHg from 1 month to 1 year, less than (70 mmHg + [2 × age]) from 1 to 10 years and <90 mmHg from 11 to 17 years}. b. Adults: systolic BP of <90 mmHg or >30% decrease from that person's baseline. PEF, peak expiratory flow; BP, blood pressure.

Therefore, anaphylaxis may occur without skin involvement, resulting in delays in recognition of anaphylaxis. Cutaneous findings of urticaria and angioedema are the most frequent manifestations (about 80–90% of anaphylaxis cases) and usually last for <24 h (72). It is important to note that urticaria is not directly related to anaphylaxis severity. Severe anaphylaxis can present without urticaria, as in some cases reports of fatal anaphylaxis (73).

Anaphylaxis, urticaria, and angioedema have similar pathogenic mechanisms, including vasodilation and increased capillary permeability. Anaphylaxis symptoms may differ according to age group. For example, children younger than 6 years are more likely to experience vomiting and cough, while older children are more likely to experience chest tightness, dizziness, hypotension, and cardiovascular collapse (18).

Different elicitors can cause distinct clinical manifestations. In perioperative anaphylaxis, cutaneous signs may not be easily seen. Urticaria and angioedema may only become apparent when the perfusion is restored, or the surgical drapes are removed (74). A study on perioperative anaphylaxis reviewed 266 reports of Grades 3–5 anaphylaxis over 1 year from all NHS hospitals in the UK. They found that the most typical presenting features were hypotension (46%), bronchospasm (18%), tachycardia (9.8%), oxygen desaturation (4.7%), bradycardia (3%), and reduced/absent capnography trace (2.3%) (75).

### When to Investigate Acute Urticaria

Current guidelines recommend that acute urticaria usually does not require a diagnostic workup because it is usually self-limiting (1, 76). Although viral or other infectious illnesses cause many cases of acute urticaria, extensive evaluation for specific viral pathogens or antiviral therapy is not indicated unless suggested by the clinical history.

The recent international European Academy of Allergology and Clinical Immunology (EAACI)/Global Allergy and Asthma European Network (GA2LEN)/European Dermatology Forum (EuroGuiDerm)/Asia Pacific Association of Allergy, Asthma and Clinical Immunology (APAAACI) guideline state that the only exception is the suspicion of acute urticaria due to a type I food allergy in sensitized patients or the existence of other eliciting factors such as non-steroidal anti-inflammatory drugs (NSAIDs) (1).

An allergic cause is possible if the clinical history suggests a specific trigger to which the patient was exposed shortly before the onset of symptoms (usually within 1–2 h after exposure). If the history does suggest a possible allergy, skin testing, serum tests for allergen-specific immunoglobulin E (IgE) antibodies are appropriate. However, the interpretation of allergy tests can require some expertise. A positive result is suggestive, although not diagnostic of allergy, and a negative result does not exclude allergy. Allergy tests and educating the patients may be helpful to allow patients to avoid re-exposure to relevant causative factors. Occasionally, it is essential to confirm a diagnosis of allergy in acute urticaria with confirmatory tests to avoid mislabeling patients as allergic. Although skin biopsy is not indicated in most cases of acute urticaria, it might occasionally help differentiate this condition from other inflammatory disorders (76).

### The Importance of Tryptase When Investigating Anaphylaxis

Tryptase is a marker of mast cell activation. It is a serine protease expressed in mast cells, and to a lesser degree, in basophils. There are four isoforms, but only α and β are considered biologically important (77).

During anaphylaxis, tryptase can be detected in serum 30 min after the onset of symptoms, peaks within 60 to 90 min, begins to decline after 2 h, and returns to normal levels within 24 to 48 h. Therefore, blood samples must be collected within 1–4 h of the reaction. Immunoassays allow detection of both total (baseline release) and mature (released only at the time of activation) tryptase. Another blood sample to measure the basal level of tryptase is needed 24 to 48 h after anaphylaxis (69, 78). In general, baseline tryptase levels >8 ng/ml are considered elevated, but this is not always a sign of mast cell activation. It is difficult to establish a cut-off point for the diagnosis. There are no special levels to confirm mast cell activation (hence anaphylactic episodes) as it must be calculated according to individual baseline tryptase levels with the formula: 1.2 x baseline + 2 ng/ml (79).

Tryptase levels are typically higher and more persistently elevated in anaphylactic reactions to intravenous drugs and insect venom than oral triggers such as food. Furthermore, elevations correlate with hypotension (69).

However, normal tryptase levels do not rule out anaphylaxis because the sensitivity is not optimal. This is explained by the fact that about 27% of the population does not have α-tryptase genes, affecting serum tryptase levels. Several studies use an equation with tryptase increasing at least 20% above baseline plus two ng/ml within 4 h of the allergic reaction (79–82). Tryptase levels ≥2 ng / mL + 1.2 × baseline is significantly increased for patients with low baseline tryptase (72, 79, 81).

Unfortunately, tryptase is not available everywhere. In a study by Jares et al. whose aim was to investigate the clinical features and management of drug-induced anaphylaxis (DIA) in Latin America, only 8 of 264 patients (3%) had tryptase levels accessed (56). In an online survey promoted by the Latin American Society of Allergy and Immunology to assess the current resources available in Latin American countries for the diagnosis and treatment of anaphylaxis, they found that the determination of serum tryptase was possible only in some health centers, often private, in five of the ten countries surveyed (83).

Differential diagnoses of elevated total tryptase levels include patients with systemic mastocytosis (SM), acute myelocytic leukemia, myelodysplastic syndromes, immunologic disorders (hypereosinophilic syndrome), severe renal failure, or familial tryptasemia – a disease associated with cutaneous flushing and pruritus, dysautonomia, functional gastrointestinal symptoms, chronic pain, and connective tissue abnormalities, due to the expression of more than two α-tryptase genes (69, 78).

### Challenges in Finding the Cause of Anaphylactic Reactions

The diagnoses of anaphylaxis should be based on relevant clinical history and a combination of available tests, i.e., skin tests, *in vitro* tests (serum tryptase, specific IgE serum levels, basophil activation test or histamine release tests) and/or provocation tests (3). However, the investigation of precipitating agents can become challenging given the complex variability in clinical presentation, multiple concurrent exposures, and many differential diagnoses, such as in the context of perioperative anaphylaxis (84).

Acute serum tryptase levels is an important tool during the diagnostic evaluation of anaphylaxis but it is not worldwide available. Moreover, it has high specificity, but low sensitivity and results should be carefully interpreted (84).

IgE-mediated anaphylactic reactions can be assessed by *in vivo* (skin tests to foods, venom, drugs, latex) and/or *in vitro* tests (serum specific IgE to foods, venom, and some drugs) (3). It is worth noting that, in the context of anaphylaxis, their detection facilitates guidance as to the allergen to be used in provocation tests. However, a positive skin test or elevated specific IgE are useful to confirm the etiology of an allergic reaction only when the clinical history is suggestive, otherwise they just reveal sensitization (84).

Most skin tests are considered safe and rapid but not free of systemic reactions. The perfect timing for performing skin tests may vary among different allergens. In general, a period of at least 4 weeks after the anaphylactic episode is suggested but could be longer for drug-induced anaphylaxis and each patient should be individually assessed. Comorbidities (e.g., asthma) must be controlled, and medications (antihistamines, high-dose corticosteroids, antidepressants, and antipsychotics with an antihistamine effect) paused prior to testing (84).

An obstacle faced when performing skin tests and determining serum specific IgE levels is to obtain cut-off values that could confirm the diagnosis and avoid provocation tests. In addition, specificity and sensitivity vary according to the trigger involved in the reaction, not being possible to accurately determine universal values of specific IgE (84).

The determination of molecular biology-based components has enabled advances in precision medicine by conferring greater specificity to diagnosis, allowing the identification of discriminative co-sensitization vs. cross-sensitization phenomena, stratifying the clinical risk associated with a specific sensitization pattern, and a better indication to the provocation test in cases of anaphylaxis by food (85–87). Molecular allergy diagnostics yielded best results in peanut and tree nut allergies (88, 89).

Despite all the scientific advancement in recent years, the provocation test is still considered the gold standard in diagnosing hypersensitivity to foods and drugs, regardless of the pathophysiological mechanism involved (85, 90). They are used to confirm, exclude, or prove tolerance to a particular food or drug and test a safe alternative (84). A significant disadvantage is a risk of inducing anaphylaxis, making provocation a high-risk procedure. The decision on its execution is influenced by clinical history, age, type of symptom, time of the last reaction, results of skin testing and/or serum levels of specific IgE, and the joint decision between physician and patient, carefully evaluating risk vs. benefit. Those with a convincing history of anaphylaxis from a specific allergen and proven evidence of specific IgE sensitization should not undergo provocation tests (87, 90).

Complementary tests, such as basophil activation test (BAT) with food, drugs, Hymenoptera venoms and latex, reflect tissue mast cell sensitization and activation. Due to the lack of standardized kits for most allergens is employed mainly in clinical research. However, BAT should be considered a diagnostic tool in selected patients, especially those with severe and high-risk anaphylaxis related to drugs (3, 90).

Further elucidation of the underlying mechanisms of anaphylaxis is needed to better characterize the phenotypes and endotypes of anaphylaxis and decrease the number of cases labeled as idiopathic anaphylaxis (3, 78).

## Acute Urticaria and Anaphylaxis Treatment

### Adrenaline and Beyond in Anaphylaxis Treatment

Epinephrine (adrenaline) is the first-line drug recommended by the American, European and World Allergy Organization guidelines for treating anaphylaxis, although its use remains suboptimal. The recommended dose is 0.01 mg/kg, maximum 0.5 mg, given intramuscularly in the mid-anterolateral region of the thigh, which can be repeated every 5–15 min as needed (3, 32, 71).

The vasodilatory effect on skeletal muscles facilitates the rapid absorption of adrenaline into the central circulation, in contrast to its vasoconstrictor effect when injected into the subcutaneous tissue, delaying its absorption and onset of action. Intravenous administration is also not recommended for initial treatment, as potentially fatal arrhythmias can occur within bolus administration of epinephrine (3, 32). However, in special circumstances such as severe hypotension, intravenous administration appears to be more effective and should be used with caution (71).

There is no absolute contraindication to the administration of epinephrine, and delays in its administration are associated with progression to severe anaphylaxis and potential death (3, 32).

As it is a non-selective agonist of all adrenergic receptors present in all organ systems affected by anaphylaxis, it exerts effects on α1 receptors causing peripheral vasoconstriction, reversing hypotension and mucosal edema; on β1 receptors increasing cardiac output, thus reversing hypotension; and on β2 receptors reversing bronchoconstriction and inhibiting the additional release of histamine and other mediators by mast cells and basophils, also preventing worsening of symptoms (3, 32, 71).

A self-injectable adrenaline device is highly recommended among experts for patients at risk of anaphylaxis (71). But despite its critical role, the self-injectable form of adrenaline is not available in most countries, being limited to only 32% of all 195 countries in the world, mainly in developed countries. The high cost is one of the main limiting factors (3).

In Brazil, for example, there is neither the manufacture nor the marketing of these devices, requiring their importation. This fact dramatically hinders the management, implementation of the “action plan,” and self-management of anaphylactic reactions outside the hospital environment (71).

Another issue that is also relevant is the expired validity of the injectors. Because they remain unused for long periods, there is a high probability that patients carry this medication with its expiration date (71). All these aspects, notably the high cost, unavailability and expired validity are barriers to the use of adrenaline autoinjectors (91).

Second-line interventions include removing the trigger when possible, calling for help, correct positioning of the patient, offering high flow oxygen, administration of intravenous fluids (crystalloids) associated with the first dose of adrenaline in patients with cardiovascular involvement and severe pictures of anaphylaxis, should also be considered. However, no robust evidence is available (3, 71).

Additionally, in cases of bronchial obstruction, inhaled short-acting beta-2 adrenergic agonists (e.g., salbutamol) can be administered. When laryngeal/pharyngeal edema has been suspected, inhaled adrenaline administration by nebulizer, as a supplement to intramuscular adrenaline, and oxygen are recommended (3, 71).

Several other drugs can be used in the additional treatment of anaphylaxis, but never in isolation since they do not have a global effect capable of reversing the systemic symptoms of anaphylaxis. The need for any additional medication should be individualized and depend on the adrenaline response (3).

Systemic antihistamines have only been shown to relieve cutaneous symptoms, and a possible effect on non-cutaneous symptoms remains unconfirmed (71). It is noteworthy that antihistamines are now a third-line treatment in some guidelines due to concerns that their administration may delay more urgent measures, such as repeated administration of adrenaline (3, 32).

Glucocorticoids are commonly used in anaphylaxis, as they are believed to prevent prolonged symptoms and possibly biphasic reactions, but there is limited evidence of their efficacy, and they may be deleterious in children; their routine use is becoming controversial (3, 71).

Parenteral administration of glucagon may be helpful in the treatment of patients with anaphylaxis refractory to adrenaline use, particularly those on beta-blocker therapy, although evidence is very limited. The dose for adults is 1–5 mg in a slow bolus, intravenously, followed by a titrated infusion of 5–15 mcg/min (3, 71).

Patients with anaphylaxis are at risk of prolonged reactions and developing biphasic reactions, although the likelihood is low. In these cases, there is a recurrence of symptoms 8 to 10 h after the initial reaction, without a new exposure to the triggering antigen, and should be treated as any anaphylaxis. Thus, more prolonged monitoring should be considered in patients with asthma, those with a history of severe anaphylaxis, biphasic reactions, and/or a need for multiple doses of adrenaline (3, 32, 71).

Education and management of anaphylaxis should be customized according to the patient's clinical history and presentation, considering their age, concomitant diseases, concomitant medications, and triggering factors (3).

### Acute Urticaria Treatment: Which Drugs and for How Long

Initial treatment of acute urticaria should focus on the short-term alleviation of pruritus and reduction of wheals. The literature on the management of acute urticaria is rare, probably because the condition is too often self-limited. Current guidelines recommend modern second-generation H1-antihistamines (such as bilastine, cetirizine, desloratadine, ebastine, fexofenadine, levocetirizine, loratadine, and rupatadine) as a first-line symptomatic treatment for acute urticaria (1, 76). The newer, second-generation H1-antihistamines are minimally or non-sedating and free of anticholinergic effects that can complicate the use of first-generation agents (92). These medications have been mostly evaluated in treating chronic urticaria (CU), and in some cases, their use in acute urticaria is extrapolated from those researches. In current guidelines, there is no recommendation on which to choose for the treatment of acute urticaria, although a few studies in patients with CU suggest that cetirizine and levocetirizine may be modestly more effective than other agents (93). Some patients require higher than standard doses to control urticaria and may experience drowsiness at those higher doses. The higher doses may have better efficacy in some adults, although this has not been conclusively demonstrated in patients with acute urticaria. Current guidelines have no specific recommendation on how long H1-antihistamines should be used in acute urticaria, but it might be required until complete symptoms are controlled (1).

First-generation antihistamines available in parenteral presentation, such as diphenhydramine and promethazine, are rapidly acting and effective in emergency units for acute urticaria treatment. However, they can be associated with sedation and impaired motor skills because of their ability to cross the blood-brain barrier in both pediatric and adult patients besides other frequent prominent anticholinergic effects, including dryness of the mouth and eyes, constipation, inhibition of micturition, and potential provocation of narrow-angle glaucoma. Thus, its use should be limited, and non-sedating 2nd generation oral antihistamines preferred as first-line treatment for most patients, especially for those with mild and moderate disease (94).

In patients with poor response to antihistamines, a brief course of oral corticosteroids might also be required while attempting to eliminate suspected triggers and develop an effective treatment plan (76). The recent international guideline recommends that for acute urticaria and acute exacerbations of CSU, a short course of oral corticosteroids limited to 10 days might be necessary to some patients (1, 23, 95). H1-antihistamine therapy should be continued during and after the course of glucocorticoids because some patients experience an exacerbation as the glucocorticoids are tapered or discontinued. If symptoms do not recur for several days after stopping glucocorticoids, then antihistamines could also be discontinued.

## Anaphylaxis Prevention - Venom Immunotherapy, Desensitization for Drugs and Foods

Prevention of anaphylaxis includes education based on the known trigger of anaphylaxis. Thus, current management relies on allergen avoidance and treatment of severe reactions with epinephrine (3). In cases of anaphylaxis by stinging insects, this can be very difficult. Also, for food allergy, avoidance of the trigger is currently the only approved therapy, and while effective, diets can be difficult to carry out (96). For drug allergies, the most common situation is to avoid the drug that caused the reaction. Food allergy and insect venom allergy present a high risk of anaphylaxis, which is unpredictable in occurrence and severity. The unpredictable nature of these allergies can affect the patient and family's psychosocial functioning and quality of life (73).

### Venom Immunotherapy (VIT)

In patients with a history of Hymenoptera sting, anaphylaxis and positive skin or *in vitro* tests (serum specific IgE) to Hymenoptera venom, venom immunotherapy (VIT) should be considered, especially in those patients with mastocytosis (97, 98).

To select VIT, it is essential to take a good clinical history. Initially, collect information about the stinging insect (i.e., number of stings, previous and subsequent re-stings, nest, extraction of the sting, death of offending insect). It is important to take information on occupational or activities linked to a higher likelihood of sting (e.g., farmers, beekeepers, outdoor sports). Furthermore, discriminate if the reaction was local or systemic. Local large reactions (LLR) are edema exceeding 10 cm, increasing within 24/48 h, and lasting longer than 72 h. Although worrisome for some patients, they have a low risk of evolution into systemic reactions (99). VIT indications are enumerated in Table 3.

**Table 3:** Indications of venom immunotherapy [adapted from (92)].

History of systemic reaction involving organs other than the skin in children and adults
In adults, systemic skin reactions with high risk of re-sting and/or compromised quality of life. In children, VIT is generally not recommended when only skin involvement is present, due to the low risk of RS after a re-sting (10%), unless the subject is at high risk for a re-sting and/or distant of emergency care facilities, and/or impaired quality of life for the patient and/or parents
Clonal mast cell disorders with a history of systemic reaction

### Desensitization for Drugs (DS)

For patients with proven or highly suspected drug hypersensitivity reaction (DHR), drug desensitization (DS) is a procedure designed to safely reintroduce drugs into patients who have had IgE/non-IgE Type I reactions (100–102).

DS is defined as the induction of a temporary state of tolerance of a drug for a hypersensitivity reaction. It is indicated in some specific situations, as shown in Table 4 (101). It is performed by administering increasing doses of the medication over a short period (from several hours to a few days) until the total cumulative therapeutic dose is achieved and tolerated (100, 102). It is a procedure that helps to prevent anaphylaxis, keeping patients in the first-line treatment and, therefore, representing an important advance in their prognosis (69). Although several protocols have been proposed to desensitize patients to different drugs, the 12-steps rapid desensitization protocol has been demonstrated to be safe and efficient and can be adapted to be used with any parenteral drug (103, 104).

**Table 4:** Drug desensitization indications [adapted from (51)].

1. When no alternative drug is available
2. When the drug involved in DHR is more effective (better quality of life; better survival) or associated with fewer adverse effects than alternative drugs
3. When the drug involved in DHR has a unique mechanism of action, like aspirin in Aspirin Exacerbated Respiratory Disease (AERD)

### Immunotherapy for Foods

Immunotherapy has several routes of administration and has been performed subcutaneously, sublingually, epicutaneously and orally. The subcutaneous approach was abandoned many years ago due to safety concerns. The sublingual and epicutaneous approaches have both been shown to be safe, but efficacy is limited by a restricted dose capacity, that is the amount that can be absorbed through the skin or under the tongue. Oral immunotherapy (OIT) is more effective than the other routes, in part because much larger doses can be administered (105).

Cow's milk, hen's egg, wheat, soy, peanut, tree nut, fish, and shellfish are most often associated with food allergies. Oral immunotherapy (OIT) is an option for individuals who do not naturally tolerate these foods by late childhood or adulthood (96).

OIT for foods involves introducing an allergenic food mixed with a vehicle in gradually increasing doses. OIT protocols include an initial escalation phase, followed by a dose build-up phase and maintenance phases. The efficacy of the OIT depends on the chosen outcomes, including the ability to tolerate the treatment, induction of a state of desensitization, and/or the development of a more durable state of clinical tolerance, what is often referred to as lack of sustained response. Adverse reactions during OIT are common. Reactions are usually mild, with local symptoms such as oral itching. However, moderate and even severe reactions may also occur, and patients may require treatment with epinephrine, especially during dose escalation (96). Recently, a death from baked milf OIT was reported in Canada, as well as an exercise-induced anaphylaxis to wheat OIT in Japan (106, 107). Eosinophilic esophagitis occurs in some patients undergoing OIT, and it is not clear how often the disease was already present before the start of OIT and could complicate the procedure (108).

In cases of idiopathic anaphylaxis, when the trigger is not known, the anti-IgE monoclonal antibody omalizumab demonstrated to be a successful treatment, effectively reducing the number of episodes, and improving quality of life (109). In addition, omalizumab has been shown to be effective as an adjunct to treatment in patients who experience episodes of anaphylaxis during immunotherapy with food (OIT) or with Hymenoptera venom (VIT). Some studies showed more safety using omalizumab in groups of patients with OIT (milk and peanut). These patients were able to tolerate a higher amount of protein with fewer reactions (110).

## Discussion

Anaphylaxis is a life-threatening reaction that requires immediate diagnosis and treatment. Anaphylactic reactions can present with a variety of symptoms, and hives and angioedema are often observed (3). On the other hand, acute urticaria is limited to the skin and mucosa and, although not potentially lethal, may impact patients' quality of life (1).

Mast cells have a central role in the pathophysiology of the two conditions, and their activation can be triggered by allergic and non-allergic mechanisms (4, 5). Many of these triggers can cause both acute urticaria and anaphylaxis, but some are more frequent in a determined region, age group or type of reaction - NSAIDs, for example, is the leading cause of drug-induced anaphylaxis in Latin America but not in the United States, and viral infections are an important cause of acute urticaria in children but not anaphylaxis (28, 56). So, it is of extreme importance to understand the potential triggers for each condition and perform an adequate investigation when recommended. In general, an extensive investigation is not necessary for acute urticaria but mandatory to search for a cause in anaphylaxis, especially to prevent future and more severe reactions (1, 3).

Acute urticaria and anaphylaxis are treated differently, at least regarding first-line therapy. Whereas H1-antihistamines are the preferred therapy in acute urticaria, their effect in anaphylaxis is limited to skin symptoms. In addition, parenteral use of antihistamines may cause hypotension as a potential side effect (111). On the other hand, adrenaline is the first drug to be administered during an anaphylactic reaction, but its use in acute urticaria should be limited for patients with moderate to severe laryngeal angioedema (1, 3).

Finally, avoiding the trigger responsible for the reaction is the best way to prevent further episodes of anaphylaxis or acute urticaria. Of course, anaphylaxis prevention is mandatory because of the risk of a severe reaction. Desensitization to drugs and foods can be an option in selected patients, as well as venom immunotherapy. In acute urticaria, preventive measures are not always possible, mainly when it is caused by virus infections or in those cases where no specific trigger can be identified.

In conclusion, reactions presenting with hives and/or angioedema must be carefully assessed to distinguish between acute urticaria or anaphylaxis, as the diagnostic investigation, treatment and preventive measures are different and can directly impact in patient's survival and quality of life.

## Author Contributions

All authors contributed to manuscript conception, design, revision, read, and approved the submitted version.

## Conflict of Interest

The authors declare that the research was conducted in the absence of any commercial or financial relationships that could be construed as a potential conflict of interest.

## Publisher's Note

All claims expressed in this article are solely those of the authors and do not necessarily represent those of their affiliated organizations, or those of the publisher, the editors and the reviewers. Any product that may be evaluated in this article, or claim that may be made by its manufacturer, is not guaranteed or endorsed by the publisher.
